# Metric System

**Published:** 1879-03-20

**Authors:** 


### *METRIC SYSTEM.*

In the course of a lecture delivered before the New York Academy of Science, to demonstrate that the metric system must be taught more through the senses than to the mind, Dr. E. Seguin shows some of the advantages physicians would derive from the use of this quantitative language in practice, and in recording observations.

"Foremost in human interest is the uniformity of weights in prescriptions, which would prevent the grave or fatal results attending the composition abroad of medicines prescribed here. And the uniformity of measures which would give the possibility of writing observations uniform—that is to say comparable at sight—with those of other nations.

"Then comes the possibility of mathematically accounting for the vital functions intrusted to the physician, in health at first, during their waste in disease, and in the course of their recuperation under treatment; and to make these individual records serve as mathematical elements of true medical statistics.

"These records and the following ones, could be rendered much more valuable by being made on *metric paper* (a paper water lined, one way with the millimeter, centimeter and decimeter-line; and the other way with centimetric and decimetric water-lines only). I will name among the applications of this paper to the precision of our art, the tracing of the curves of the diurnal and pathological variations of the temperature, pulse and respiration; the figures furnished by the hæmatometers, spyrometer, dynamometer, various urinoscopes, etc.; the graphic of the myograph, of the sphygmograph, and of other self-graphing instruments; the drawings or photographs of the microscopic specimens, and other perishable or evanescent data, which lose part of their interest if their proportions have not been taken on an invariable scale.

"I cannot omit the use of the metric paper-bands, on which, in important cases, must be telegraphed, from the bedside to the doctor's desk, the sudden break in the course of the vital functions, which foretells a crisis avertable only by telegraphic swiftness. Now that instruments of medical precision are invented as fast as the want of nicety in diagnosis demands them, nothing is more necessary than a means of concentrating their data on the uniform, and all but uniformly accepted plans of the metric system, of the metric scales, and of the metric paper. The great founders of the first societies of 'medical observation,' longed in vain for such means of record of their admirable observations as are now within the grasp of the whole profession.

"Let us, therefore, follow Europe in the use of the metric system; but let us show European physicians the use of the 'metric plan' in medical observation, beating them on their own ground, literally with a piece of paper.

"Our mind has come to that, but not our routine and automatism.

Most of our medical schools and hospital clinics teach by the old weights and measures; few physicians prescribe in the metric language, and the druggists who know better (not all, of course), smile and put up according to direction. The best surgeons import their instruments for no other superiority over our home manufactures than their metric scale. The Massachusetts, Pennsylvania and New York State Medical Societies recommend the use of the metric system; the U. S. Medical Marine Service makes it official and obligatory; and the Secretary of the Navy sarcastically remarks, in his last report to Congress, that this change has caused no evil results. Could we not benignantly promise the same results to private initiative?"—*Medical Record.*

